# Properties of Al_x_Ga_1−x_As grown from a mixed Ga–Bi melt

**DOI:** 10.1038/s41598-024-51234-0

**Published:** 2024-01-16

**Authors:** Olga Khvostikova, Alexey Vlasov, Boris Ber, Roman Salii, Vladimir Khvostikov

**Affiliations:** Ioffe Institute, Politechnicheskaya 26, St. Petersburg, Russia 194021

**Keywords:** Materials for optics, Metals and alloys, Materials for devices, Electronic devices

## Abstract

Thick smoothly graded Al_x_Ga_1−x_As layers (50–100 µm) are used in light-emitting diode structures and also for creation of high-power photovoltaic converters with side-input of laser radiation. To achieve the required thickness of the Al_x_Ga_1−x_As layer the high temperature LPE growth technique is required. However high epitaxial temperature increases the unintentional doping level. Epitaxy from mixed Ga–Bi melts was investigated as a way to solve this problem. It was found that for growing relatively thick Al_x_Ga_1−x_As layers, it is expedient to use Ga–Bi melts with 20 at% or less bismuth content. SIMS and Hall characterization of Al_x_Ga_1−x_As layers revealed that the growth of Al_x_Ga_1−x_As from mixed Ga-Bi melts reduces the background doping level (including carbon) and influences the native defect formation keeping the n-type conductivity. This effect is explained by the changes of the group III and V elements concentrations in the melt as well as Bi incorporation in the lattice.

## Introduction

In recent years, research efforts have been carried out to create laser and solar power converters with side-input of light^1,2^. A distinctive feature of AlGaAs/GaAs based photovoltaic converters (PVC) is the presence of a thick Al_x_Ga_1−x_As layer (more than 50 μm) with a gradient composition^3–6^. In contrast to Si- and GaAs-based devices, owing to the gradient refractive index, AlGaAs layer helps to collect the light in the active area. Similar layers can also be used in LED structures^7,8^.

Thick layers can be grown cost-effectively by liquid phase epitaxy (LPE). The growth of thick Al_x_Ga_1−x_As layers (more than 50 µm, x = 0.55–0.1) requires high initial epitaxy temperatures (850–900 °C)^9–11^. This is especially important for solid solution compositions with x > 0.35, where the layer growth rate drops ~ 1.5–2 times^11,12^. The probability of background doping increases at high temperatures. For example, undoped AlGaAs layers grown from a Ga melt at T = 850 °C reveal p-type conductivity with a concentration of (4–5)∙10^16^ cm^−3^^13^. The problem of conductivity type inversion (from n to p- type) is observed for undoped GaAs grown at T > 850 °C from a Ga melt^14,15^. At the same time, layers grown from Bi-melts at T = 700–900 °C were n-type. The inversion of the conductivity type during the growth from Ga melts at high temperatures can be explained by a large number of acceptor centers: V_As_, Ga_As_, or C_As_. The growth from the Bi melt changes the ratios of gallium and arsenic in the liquid. This decreases the concentration of native defects in GaAs (V_As_ mainly) and influences the distribution coefficient of background impurities^14–16^. GaAs grown from mixed Ga-Bi melts was found to have the minimum carbon content at x_Bi_ = 25–50 at% in the melt^17^.

Bi is not a traditional element for the III-V materials (e.g. GaBi has not yet been synthesized in the crystalline form^18^). There have been several studies during last decade demonstrating the growth of GaAs_1−x_Bi_x_ alloys with x up to ~ 20%^19^. The growth of such alloys is possible in non-equilibrium thermodynamic conditions at low temperatures and commonly is provided by the low temperature MBE (molecular beam epitaxy). More often Bi is used as a surfactant in conventional MBE or MOVPE (metalorganic vapour-phase epitaxy) growth, where it changes the surface reconstruction and/or surface free energy influencing the growth processes^20,21^. At the same time in thermodynamic equilibrium conditions (which is the case of LPE) Bi is characterized by a very low solid solubility in III–V compounds due to the large atomic size. In GaAs the equilibrium solid solubility is reported to stay below (1–6)·10^18^ cm^−3^^22,23^, i.e. no solid solution can be formed.

**Figure 1:**
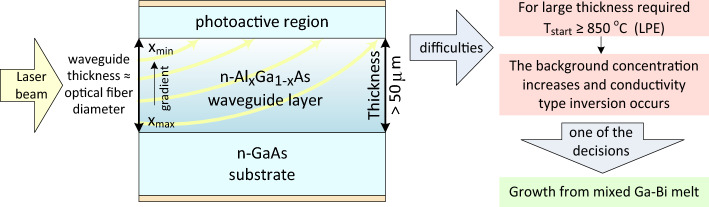
Scheme of side-input photoconverter with thick gradient AlGaAs layer.

To date, the properties of Al_x_Ga_1−x_As layers grown from a Bi-containing melt have not been studied. Phase diagrams of Al–Ga–As–Bi system at T ~ 900 °C were modeled only for the case of the Bi-enriched melt (x^L^_Ga_ ≤ 10 at%)^24^. The use of mixed Ga–Bi melts for the crystallization of Al_x_Ga_1−x_As layers at high temperature is promising from the point of view of reducing the level of background doping and maintaining the n-type conductivity of the layer (Fig. 1). Below we present novel research of the growth parameters and properties of Al_x_Ga_1−x_As layers grown from mixed Ga-Bi melts (x^L^_Ga_ ~ 50–95 at%).

## Methods

### Growth technique

**Figure 2:**
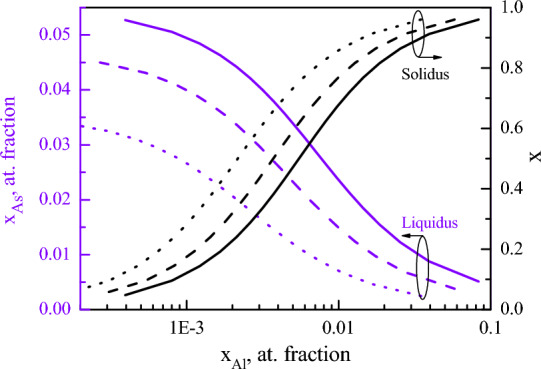
Liquidus-solidus isotherms of Al-Ga-As-Bi system for various bismuth content in the melt (solid curve—x_Bi_ = 10 at%, dash curve—x_Bi_ = 30 at%, dot curve—x_Bi_ = 50 at%).

AlGaAs layers were grown by liquid phase epitaxy in a piston graphite boat. The melt height is constant in this design of the boat, which reduces the error in determining the thickness of the layer or the growth rate ^25,26^. Theoretical liquidus and solidus isotherms (Fig. 2) were calculated based on the quasi-regular solutions model at T = 900 °C^27^ and interaction parameters^28^. The calculated curves are confirmed by experimental data^29^.

The growth process took place in a quartz reactor in a flow of purified hydrogen. The temperature was measured by a Pt–Pt/Rh-thermocouple and controlled with an accuracy of ± 1 °C. The purity of gallium was 5N and bismuth—6N. The melts were homogenized at 920 °C for an hour. The start temperature of epitaxy was 900 °C with the cooling rate—0.9 °C/min.

### Characterization

Hall measurements were carried out by the four-probe van der Pauw method on an Ecopia HMS-3000 setup. Indium (In) contacts to n-Al_x_Ga_1−x_As were annealed at 350–420 °C. For Hall measurements layers were grown on a semi-insulating GaAs (SI) substrate at 900 °C from melts with various bismuth contents (x^L^_Bi_ = 1–50 at%). In the composition range near the intersection point of Γ, L and X valleys (x ~ 0.41) the Hall mobility correlates with the drift mobility through the Hall factor r ≠ 1 (due to the inter-valley scattering process^30^), therefore we have chosen composition x = 0.2–0.24 for the comparative study. The thickness of the grown layers did not exceed 6 μm and, therefore, the solid solution composition remained fixed. The layer thickness was controlled by an optical microscope. To highlight the heterointerface, the samples were anodically oxidized in an acid–glycol–water solution. Since GaAs and AlGaAs have different oxidation constants, the colors of the layer and the substrate were different^31^.

It is known that semi-insulating GaAs also changes the conductivity type to p-type at temperatures above 900 °C^32^. This also happened in our case in the graphite boat at T ≥ 900 °C during the melt homogenization period. To avoid uncertainty errors during Hall measurements, prior to the layer growth the substrate was etched (3–6 s) with an arsenic-unsaturated melt in order to remove the conductive surface layer. The AlGaAs layers were not intentionally doped, except for the tin originated from GaAs:Sn used for the saturation of the melt. The content of tin in the melt was less than 10^–6^ at. fractions, which is a background level.

The content of carbon and bismuth impurities in AlGaAs layers was studied by dynamic secondary ion mass spectrometry (D-SIMS) using an IMS 7f secondary ion microprobe (CAMESA, France). ^133^Cs^+^ ions were used as primaries with the impact energy of 15 keV and the beam current of 100 nA. The primary beam was rastered over the area of 130 × 130 μm^2^. The negative ions ^12^C^−^, ^209^Bi^−^ and ^75^As^−^ were used as secondary (analytical) ions for determining the content of impurities. The composition of the layer was controlled using the negative secondary ions ^27^Al^−^ and ^69^Ga^−^. To suppress the influence of the atoms sputtered from the walls of the sputtering crater, the diameter of the analyzed area was set to 33 μm for measuring the carbon content and to 60 μm for bismuth. When measuring carbon impurity the energy window of the spectrometer was fully opened (140 eV) and the mass resolution was set to M/ΔM = 400. The pressure in the analytical chamber of the mass spectrometer during the measurements was 4 × 10^–10^ Torr. To reduce the carbon detection limit, the samples were kept in the analytical vacuum chamber for three days.

When measuring bismuth in substances containing gallium, we should take into account the possible superposition of analytical signals from the secondary ions ^209^Bi^−^ and ^69^Ga^69^Ga^71^Ga^−^ molecular ions with similar masses. To exclude the superposition of these signals, the measurements were carried out with the mass resolution M/ΔM = 2000 at the secondary ion energy window width of 60 eV.

Quantitative analysis of the carbon and bismuth content in the layers was carried out according to a procedure based on relative sensitivity factors (RSF)^33,34^. To study the carbon content, the RSF was preliminarily determined using the reference samples of GaAs and AlGaAs layers that were implanted carbon at the dose of 1 × 10^15^ cm^−2^ and the energy of 120 keV. The bismuth content was estimated using its relative sensitivity factors for the GaAs matrix^35^.

The photoluminescence (PL) spectra were recorded with a liquid nitrogen cooled cryostat and a single channel detector (photomultiplier). The 532 nm CW laser was used for excitation.

## Results and discussion

Growth rate is an important parameter during crystallization. In our case growth rate depends mainly on the slope of the liquidus, because the melt thickness stayed the same (1.5 mm above the substrate) and the growth was carried out at the same cooling rate of 0.9 °C/min. The liquidus slope (ΔT/Δx_As_) at the fixed temperature range (ΔT = const) depends on arsenic solubility x_As_. As can be seen from Fig. 2, the growth rate will reduce when bismuth concentration is increased in the liquid due to lower x_As_ values. The diffusion coefficient of arsenic in the mixed Ga-Bi melt will also influence the growth rate. Since the diffusion coefficient is inversely proportional to the viscosity of the liquid^36^ (and the viscosity of bismuth is ~ 2 times higher than that of gallium^37,38^), the growth rate will depend on the content of bismuth in the mixed Ga–Bi melt.

**Figure 3:**
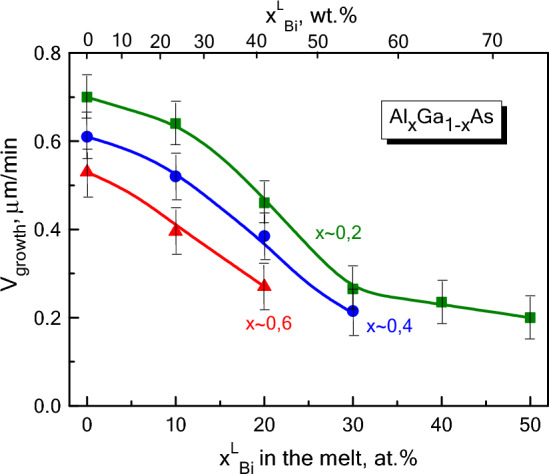
Dependence of the growth rate of Al_x_Ga_1-x_As layer on the bismuth content in the liquid phase for various compositions of the solid solution.

Biryulin et al.^16^ studied the mechanism of GaAs «purification» with bismuth and provided an estimation of the arsenic diffusion coefficient in a mixed Ga-Bi melt at T = 800 °C. These values differ significantly in gallium D_As_ ~ (1.0–1.2)∙10^–5^ cm^2^/s and bismuth D_As_ ~ (0.4–0.6)∙10^–5^ cm^2^/s melts. The authors suggested that mass transfer in the liquid phase at different bismuth content (x_Bi_) is limited by different processes. The Ga-Bi binary system is characterized by an immiscibility region up to 262 °C (8.5 < x_Bi_ < 61.5 at%). However, according to^16^, at 800 °C there is a noticeable correlation in the spatial arrangement of Ga and Bi atoms in the liquid (the density of Bi is 1.7 times greater than that of Ga). At moderate contents of bismuth (up to 25 at% ≈ 50 wt%) the arsenic mass transfer to the growth boundary is determined mainly by fast diffusion processes along “gallium” channels. When the weight fraction of bismuth becomes greater, the arsenic mass transfer switches to slow diffusion processes along “bismuth” channels. This explains a sharp decrease in the layer growth rate at x_Bi_ > 20 at%. This tendency was verified experimentally for Al_x_Ga_1−x_As layers of different composition (x) grown at T = 900 °C (Fig. 3).

The main background impurity in liquid-phase epitaxy with a quartz reactor and a graphite boat is carbon, which plays a role of a p-type dopant^39^. The aim of reducing carbon content becomes especially important when growing thick gradient AlGaAs structures at high temperatures T > 850 °C. Carbon is known to have a low vapor pressure^40^, and melt annealing does not lead to a decrease in its concentration^15^. Our observations of undoped AlGaAs layers grown from Ga melts at T = 900 °C revealed p-type conductivity with p = (3–6)∙10^16^ cm^−3^. Like GaAs^14–17,41^, bismuth seems to be the key to solving the problem of carbon in AlGaAs.

**Figure 4:**
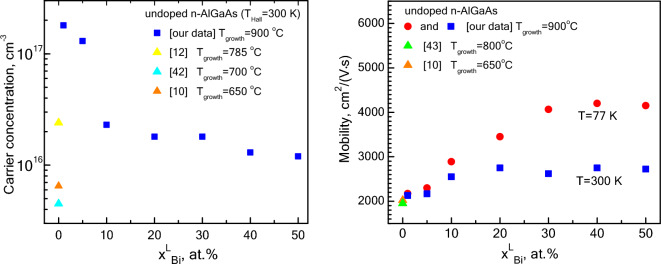
Hall data of Al_x_Ga_1-x_As layer (x = 0.2–0.24) grown from Ga-Bi-melt with various Bi content in the liquid phase and reported data (triangles, Ga-melt). Circles—T_Hall_ = 77 K, squares and triangles—T_Hall_ = 300 K.

Figure 4 presents the results of the Hall measurements for Al_x_Ga_1−x_As (x = 0.20–0.24) layers grown from mixed Ga-Bi melts with different bismuth content (x_Bi_ = 1–50 at%). All layers grown from mixed melts possessed n-type conductivity. An increase in x_Bi_ in the melt from 1 to 10 at% leads to a decrease in the carrier concentration by an order of magnitude and an increase in the carrier mobility. This means that the background doping level (including native defects) gradually decreases. Further increase of bismuth content in the melt (20 at% and more) does not influence the concentration and mobility of electrons that much. At low bismuth content in the melt (x_Bi_ < 10 at%) the low temperature mobility values are close to the room temperature ones indicating the overcompensated character of the AlGaAs alloy. At higher Bi content (x_Bi_ ≥ 10 at%) the electron concentration and room temperature mobility are stabilized, but low temperature mobility grows, evidencing the lowering of the total background impurities concentration. For a comparison we indicated in Fig. 4 some Hall data values reported earlier for AlGaAs grown by LPE^10,12,42,43^. It can be seen that layers grown from Ga melt demonstrate lower electron concentrations at lower growth temperature. Higher growth temperatures (above 850 °C) result in p-type conductivity and cannot be compared. Thus it can be concluded that bismuth containing melts are reducing the background doping level restoring the “natural” n-type conductivity.

**Figure 5:**
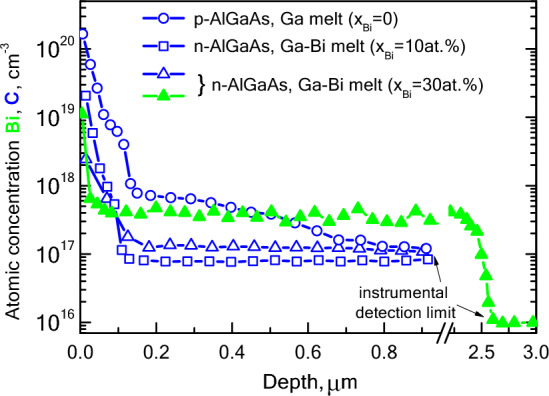
SIMS profiles of carbon (blue, open symbols) and bismuth (green, solid symbols) in Al_x_Ga_1−x_As layer (x = 0.2) with various Bi content in liquid phase (x_Bi_).

For a more detailed study of the changes in the background doping level, the content of carbon, a major background acceptor impurity in Al_x_Ga_1−x_As layers, was analyzed by SIMS. Figure 5 shows the carbon SIMS profiles in the Al_x_Ga_1−x_As structure grown from Ga melt (x_Bi_ = 0) and mixed Ga-Bi melt (x_Bi_ = 10 and 30 at. %). A significant decrease in the surface concentration of carbon can be noticed when bismuth concentration increases. The carbon containing layers are 0.1–0.12 µm thick.

The high content of carbon in the sub-surface region can be explained by the mechanism of its incorporation into the crystal structure. Major intrinsic defects in AlGaAs are arsenic vacancies, which in the presence of an excess of gallium (growth from a Ga-enriched melt) give rise to Ga_As_—acceptor antisite defects (here we entitle group-III sublattice). The arsenic vacancies are occupied by carbon V_As_ → C_As_—an acceptor^44^. The growth from Bi-containing melts gives an advantage in reducing gallium concentration in the liquid phase, leading to a decrease in the concentration of acceptor centers V_As_, Ga_As_, and C_As_.

The SIMS method revealed bismuth atoms in the layers grown from the Bi-containing melts. Bi concentration ranged from 3·10^17^ to 8·10^17^ cm^−3^. An example of a Bi concentration profile is shown in Fig. 5. These concentration values are estimates, as they were obtained with GaAs:Bi reference samples, although they demonstrate the presence of bismuth and the correlation of its concentration in the solid and liquid phases.

**Figure 6:**
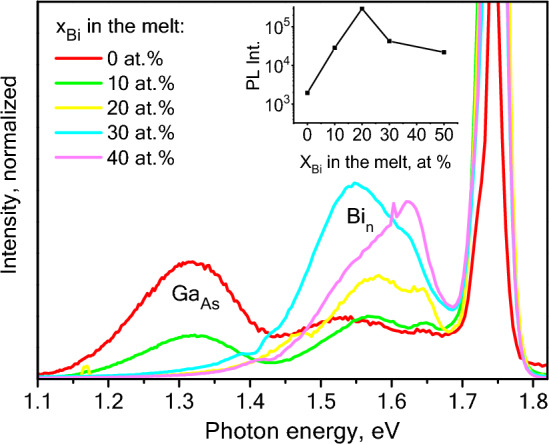
Photoluminescence spectra (77K) of Al_x_Ga_1−x_As (x ~ 0.2) grown from the Ga–Bi melt with various Bi content. CW power density ~ 10W/cm^2^.

Figure 6 presents 77 K PL spectra of the samples grown from bismuth containing melts. The LPE method does not allow precise control of the alloy composition (especially in the selected temperature range), therefore, the spectra were shifted slightly to match the interband transitions (just to visualize the subbandgap transitions energy shift). The spectra were measured at low pumping density ~ 10W/cm^2^ for better visualization of the deep level transitions. It can be noticed that the deep levels in the alloy undergo significant changes. For example, a sample grown from a bismuth-free melt reveals an intense signal of donor–acceptor transitions: the so-called EL2 and EL5 bands, traditionally associated with V_Ga_ and Ga_As_ defects. A small (10 at.%) addition of bismuth to the melt leads to a significant decrease in the EL2 and EL5 bands intensity. 20 at.% or more bismuth concentration in the melt results in the complete disappearance of these bands. This process is accompanied by a cascade of transitions with 0.12–0.36 eV energies. Similar phenomena are observed in dilute GaAsBi alloys and are identified as bismuth complexes whose energy levels depend on the number of Bi atoms in the complex^45,46^.

Photoluminescence spectra provide only qualitative information on the deep levels. Bismuth also causes changes in the surface recombination rate. Taking into account the fact that the samples were grown under the same conditions, there are no grounds to expect any significant change in the concentration of nonradiative recombination centers (first of all, oxygen atoms), but the photoluminescence spectra demonstrate a significant (up to 100-fold) increase in intensity for the samples grown from bismuth containing the melt (insert in Fig. 6). Since the PL measurements were made at a low pumping density, surface recombination and nonradiative defect recombination play the most important role in the observed intensity and, therefore, it can be assumed that the use of bismuth-containing melts leads to a significant slowdown of these mechanisms.

According to estimations in^15^ we can conclude that the decrease in acceptor centers V_As_, Ga_As_, and C_As_ in AlGaAs layers occurs due to isovalent doping with bismuth, since its activity in the liquid phase increases and the reaction of bismuth incorporation in the lattice on free arsenic sites becomes more preferable than that of gallium or carbon.

## Conclusion

Our results lead us to the conclusion that the background doping level in LPE is determined mainly by the processes occurring at the interface between the liquid and solid phases and the amount of vacant places in the lattice (namely V_As_) governs the impurity incorporation (especially carbon). The «purification» effect is observed with the use of mixed Ga-Bi melts. The growth from Bi-containing melts results in a lower concentration of acceptors (C_As_ and Ga_As_). Al_x_Ga_1−x_As layers change the conductivity from p-type to n-type starting from x_Bi_ = 1at%. It has been established that the growth rate from melts with x_Bi_ > 20 at. % decreases by over 2 times, which makes these melt compositions unsuitable for obtaining thick (more than 50 μm) Al_x_Ga_1−x_As layers. The results of this study can be useful for creating optoelectronic devices with an AlGaAs layer using a high growth temperature.

## Data Availability

All the data generated or analyzed during this study are included in this manuscript.
